# Anatomy Ontology Matching Using Markov Logic Networks

**DOI:** 10.1155/2016/1010946

**Published:** 2016-06-13

**Authors:** Chunhua Li, Pengpeng Zhao, Jian Wu, Zhiming Cui

**Affiliations:** School of Computer Science and Technology, Soochow University, Suzhou 215006, China

## Abstract

The anatomy of model species is described in ontologies, which are used to standardize the annotations of experimental data, such as gene expression patterns. To compare such data between species, we need to establish relationships between ontologies describing different species. Ontology matching is a kind of solutions to find semantic correspondences between entities of different ontologies. Markov logic networks which unify probabilistic graphical model and first-order logic provide an excellent framework for ontology matching. We combine several different matching strategies through first-order logic formulas according to the structure of anatomy ontologies. Experiments on the adult mouse anatomy and the human anatomy have demonstrated the effectiveness of proposed approach in terms of the quality of result alignment.

## 1. Introduction

Ontological techniques have been widely applied to medical and biological research [1]. The anatomy of model species is described in ontologies, which are used to standardize the annotations of experimental data, such as gene expression patterns. Such ontologies of anatomy and development facilitate the organization of functional data pertaining to a species. To compare such data between species, we need to establish relationships between ontologies describing different species [2]. For example, all gene expression patterns described in ZFIN (the Zebrafish Model Organism Database) are annotated using the zebrafish anatomy ontology. A list of such ontologies is kept on the Open Biomedical Ontologies (OBO) website [3].

Heterogeneity is an inherent characteristic of ontologies developed by different parties for the same (or similar) domains. Semantic heterogeneity has become one of the main obstacles to sharing and interoperation among heterogeneous ontologies. Ontology matching, which finds semantic correspondences between entities of different ontologies, is a kind of solutions to the semantic heterogeneity problem [4]. The matching techniques can be classified in a first level as element-level techniques and structure-level techniques. Element-level techniques obtain the correspondences by considering the entities in the ontologies in isolation, therefore ignoring that they are part of the structure of the ontology. Structure-level techniques obtain the correspondences by analyzing how the entities fit in the structure of the ontology [5].

Recently, probabilistic approaches to ontology matching which compare ontology entities in a global way have produced competitive matching result [6–9]. OMEN [6] was the first approach that uses a probabilistic representation of ontology mapping rules and probabilistic inference to improve the quality of existing ontology mappings. It uses a Bayesian net to represent the influences between potential concept mappings across ontologies. Based on OMEN, Albagli et al. [7] introduced a novel probabilistic scheme iMatch for ontology matching by using Markov networks rather than Bayesian networks with several improvements. The iMatch better supports the noncausal nature of the dependencies for using undirected networks. Niepert et al. [8] presented a probabilistic-logical framework for ontology matching based on Markov logic. Markov logic has several advantages over existing matching approach and provides a unified syntax that supports different matching strategies in the same language. Li et al. [9] improve the Markov logic model with match propagation strategy and user feedback. References [8, 9] have shown the effectiveness of Markov logic model on conference datasets.

In this paper, we consider the Markov logic based framework for anatomy ontology matching. We combine several different matching strategies through first-order logic formulas according to the structure of anatomy ontologies.

## 2. Materials

To evaluate the performance of our proposed approach, we conduct an experimental study using the adult mouse anatomy (2744 classes) and the NCI Thesaurus (3304 classes) describing the human anatomy, which are large and carefully designed ontologies. They also differ from other ontologies with respect to the use of specific annotations and roles, for example, the extensive use of the* part*_*of* relation. The two resources are part of the Open Biomedical Ontologies (OBO) [3]. We download the owl version of the two ontologies and the reference alignment (with 1516 correspondences) from OAEI anatomy track [10].

NCI Thesaurus published by the National Cancer Institute (NCI) contains the working terminology of many data systems in use at NCI. Its scope is broad as it covers vocabulary for clinical care as well as translational and basic research. Among its 37,386 concepts, 4,410 (11.8%) correspond to anatomical entities (anatomic structure, system, or substance hierarchy). Adult mouse anatomy ontology has been developed as part of the mouse Gene Expression Database (GXD) project to provide standardized nomenclature for anatomical entities in the postnatal mouse. It will be used to annotate and integrate different types of data pertinent to anatomy, such as gene expression patterns and phenotype information, which will contribute to an integrated description of biological phenomena in the mouse [11].

## 3. Methods

In this section, we present our Markov logic model for anatomy ontology matching. Our model deviates from [8, 9] in several important ways. First, we model the important hierarchy structure defined by the property of part_of, while previous works consider only subclass-superclass hierarchy. In contrast, our model does not model property correspondences for there are few properties definitions in anatomy ontologies. Another difference is in computing a priori similarities. For conference data sets, [8, 9] apply a similarity measure on the name of matchable entities. However, the class name in anatomy ontology is meaningless signature such as “NCI_C12877.” Therefore, we apply a similarity measure on the labels of classes.

**Figure 1:**
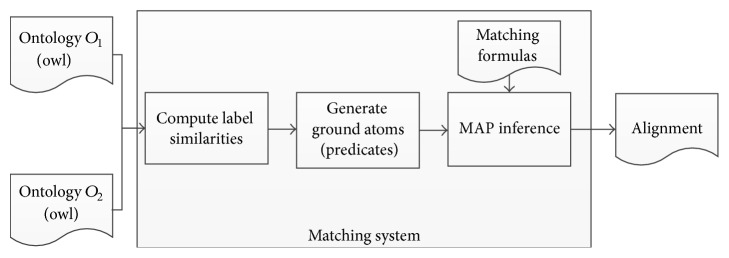
Matching system architecture.

We compute an alignment for anatomy ontologies through the following three steps. First, we compute a priori similarity based on Levenshtein distance between labels of two classes from different ontologies and apply a threshold to generate candidate matches. Then, we convert the representation of input ontologies to first-order logic predicate and define a set of formulas as matching strategy. Finally, we execute MAP inference in generated Markov networks as alignment process and output the optimal alignment. Our matching system architecture based on Markov logic networks is illustrated in Figure 1.

### 3.1. Markov Logic Networks

Markov logic networks [12] is a statistical relational learning language based on first-order logic and Markov networks. A set of formulas in first-order logic can be seen as a set of hard constraints on the set of possible worlds: if a world violates even one formula, it has zero probability. The basic idea in Markov logic is to soften these constraints: when a world violates one formula it is less probable, but not impossible. The fewer formulas a world violates, the more probable it is. Each formula has an associated weight that reflects how strong a constraint it is: the higher the weight, the greater the difference in log probability between a world that satisfies the formula and one that does not, other things being equal.

**Definition 1 .**

A Markov logic network *L* is a set of pairs (*F*_*i*_, *w*_*i*_), where *F*_*i*_ is a formula in first-order logic and *w*_*i*_ is a real number. Together with a finite set of constants *C* = {*c*_1_, *c*_2_,…, *c*_|*C*|_}, it defines a Markov network *M*_*L*,*C*_ as follows:

(1) *M*_*L*,*C*_ contains one binary node for each possible grounding of each predicate appearing in *L*. The value of the node is 1 if the ground atom is true and 0 otherwise.
(2) *M*_*L*,*C*_ contains one feature for each possible grounding of each formula *F*_*i*_ in *L*. The value of this feature is 1 if the ground formula is true and 0 otherwise. The weight of the feature is *w*_*i*_ associated with *F*_*i*_ in *L*.

An MLN can be viewed as a template for constructing Markov networks. Given different sets of constants, it will produce different networks, but all will have certain regularities in structure and parameters, given by the MLN (e.g., all groundings of the same formula will have the same weight). We call each of these networks a ground Markov network to distinguish it from the first-order MLN. From Definition 1, the probability distribution over possible worlds *x* specified by the ground Markov network *M*_*L*,*C*_ is given by

(1) $$\begin{array}{c} P\left(\;X=x\;\right)=\frac{1}{Z}\exp\left(\;\underset{i}{\sum }\omega_{i}n_{i}\left(\;x\;\right)\;\right)=\frac{1}{Z}\underset{i}{\prod }\phi_{i}{\left(\;x_{\left\{i\right\}}\;\right)}^{n_{i}\left(x\right)}, \end{array}$$

where *n*_*i*_(*x*) is the number of true groundings of *F*_*i*_ in *x*, *x*_{*i*}_ is the state (true values) of the atoms appearing in *F*_*i*_, and *ϕ*_*i*_(*x*_{*i*}_) = *e* ^*ω*_*i*_^.

In the context of ontology matching, possible worlds correspond to possible alignment and the goal is to determine the most probable alignment given the evidence. It was shown that Markov logic provides an excellent framework for ontology matching as it captures both hard logical axioms and soft uncertain statements about potential correspondences between ontological entities.

### 3.2. Ontology Representation

An ontology specifies a conceptualization of a domain in terms of classes and properties and consists of a set of axioms. Matching is the process of finding relationships or correspondences between entities from different ontologies. An alignment is a set of correspondences. A correspondence is a triple 〈*e*, *e*′, *r*〉 asserting that the relation *r* holds between the ontology entities *e* and *e*′, where *e* is an entity from ontology *O* and *e*′ is an entity from ontology *O*′ [4]. The generic form of correspondence captures a wide range of correspondences by varying what is admissible as matchable element and semantic relation, for example, equivalence ( = ), more general (⊒). In the following we are only interested in equivalence correspondence between classes across anatomy ontologies.

**Figure 2:**
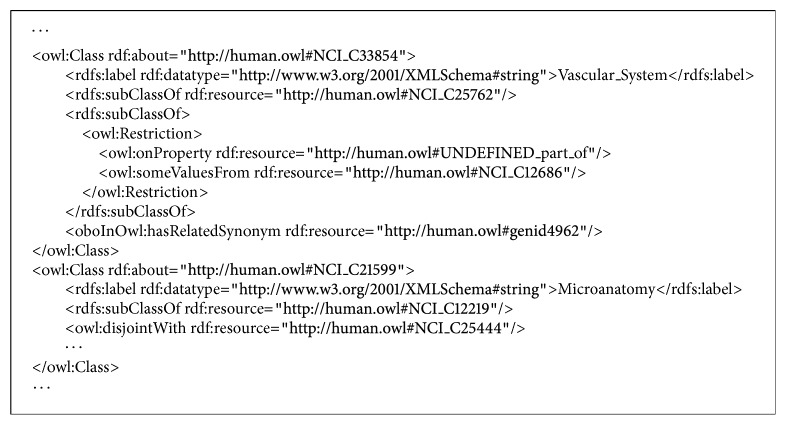
Example ontology fragments from the human anatomy ontology.

The two input ontologies are described in OWL (Web Ontology Language).* Classes* are concepts organized in a* subclass-superclass* hierarchy with multiple inheritances. The properties of* is*_*a* and* part*_*of* describe the part and whole relationship between two classes. The properties of* disjointWith* describes relationship between two classes which is interpreted as the emptiness of the intersection of their interpretations. For example, in OWL we can say that Plant and Animal are disjoint classes: no individual can be both a plant and an animal (which would have the unfortunate consequence of making SlimeMold an empty class). SaltwaterFish might be the intersection of Fish and the class SeaDwellers.  Figure 2 depicts fragments of human and mouse anatomy ontologies.

We introduce a set of predicates to model the structure of ontologies to be matched. The defined predicates are shown in Table 1. We use predicate *class*_*i*_ to represent a class from ontology *O*_*i*_. For example, *class*_1_(“NCI_C33854”) representing “NCI_C33854” is a class from ontology *O*_1_. We use predicate *sub*_*i*_ and *part*_*i*_ to model the class hierarchy in ontology *O*_*i*_, for example, *sub*_1_(“NCI_C33854”, “NCI_C25762”) and *part*_1_(“NCI_C33854”, “NCI_C12686”). The predicate *dis*_*i*_ models the disjointness relationship between two classes, for example, *dis*_1_(“NCI_C21599”, “NCI_C25444”). The predicate *label*_1_(“NCI_C33854”, “Vascular_System”) represents class “NCI_C33854” with label “Vascular_System.” We also propose a predicate *sim* to represent the similarity between labels of two classes from different ontologies, for example, *sim*(“Vascular_Endothelium”, “blood vessel endothelium”, *σ*), where *σ* is a real number. If we apply a similarity measure based on the Levenshtein distance [13], we have *σ* (“Vascular_Endothelium,” “blood vessel endothelium”) equal to 0.54. The application of a threshold *τ* is a standard technique in ontology matching. We only generate ground atoms of *sim* for those pairs of labels whose similarity is greater than *τ*. Correspondences with a similarity less than *τ* are deemed incorrect.

We differentiate between two types of predicates: hidden and observed. The ground atoms of observed predicates are seen and describe the knowledge encoded in the ontologies. The ground atoms of hidden predicates are not seen and have to be predicted using MAP inference. We use hidden predicates *map* to model the sought-after class correspondences.

We use the following notation conventions in Table 1 and through the rest of this paper:

(1) All entities from ontology *O*_1_ have a subscript “1”; all entities from ontology *O*_2_ have a subscript “2.”
(2) Lowercase *a*, *b*, and *c* with or without a subscript are a class.
(3) Lowercase *l* with or without a subscript is a label.

### 3.3. Matching Formulas

With predicates defined, we can now go on to incorporate our strategies about the task using weighted first-order logic formulas. Markov logic combines both hard and soft first-order formulas. This allows the inclusion of both known logical statements and uncertain formulas modeling potential correspondences and structural properties of the ontologies. Then it makes joint inference of two and more interdependent hidden predicates.

We will introduce five types of constraints to model different matching strategies, namely, a priori confidences, cardinality constraints, coherence constraints, stability constraints, and match propagation. The formula without a weight is a hard constraint and holds in every computed alignment. The formula with a weight is a soft constraint and the weight reflects how strong a constraint it is. For simplicity, we will from now on assume that the predicate *class*_*i*_ is implicitly added as a precondition to every formula for each class appearing in the formula.

*A Priori Confidences*. We compute an initial a priori similarity *σ* for each pair of labels of two classes across ontologies based on the Levenshtein distance [13] and use a cut-off threshold *τ* to produce matching candidates, above which ground atoms of predicates *sim* are added to the ground Markov network. The higher the similarity between labels of two classes is, the more likely the correspondence between the two classes is correct. We introduce the following formula to model the a priori confidences of a correspondence:

 σ  
  (2) label1c1,l1∧label2c2,l2∧siml1,l2,σ=>mapc1,c2.

Here, we use the similarity *σ* between labels as the formula weight since the confidence of a correspondence to be correct depends on how similar their labels are.

*Cardinality Constraints*. In general, alignments can be of various cardinalities: 1 : 1 (one to one), 1 : m (one to many), n : 1 (many to one), and m : n (many to many). In this work, we assume the one to one constraint. We use two hard formulas stating that one concept from ontology *O*_1_ can be equivalent to at most one concept in ontology *O*_2_, which ensures the consistency of a computed alignment and vice versa:

(3) mapa1,a2∧mapa1,b2=>a2=b2mapa1,a2∧mapb1,a2=>a1=b1.

*Coherence Constraints*. Coherence constraints reduce incoherence during the alignment process. These constraints formulas are added as hard formulas to ensure satisfaction in the computed result alignment. The following formulas describe that two disjoint classes of ontology *O*_1_ will not match two classes of ontology *O*_2_ with subclass relationship respective simultaneously and vice versa:

(4) sub1a1,b1∧dis2a2,b2=>!mapa1,a2∧mapb1,b2dis1a1,b1∧sub2a2,b2=>!mapa1,a2∧mapb1,b2.

*Stability Constraints*. The idea of stability constraints is that an alignment should not introduce new structural knowledge. The formulas for stability constraints are soft formulas associated with weights reflecting how strong the constraints are. When an alignment violates one soft formula it is less probable, but not impossible. Formulas (5) and (6) decrease the probability of alignments that map concept *a*_1_ to *a*_2_ and *b*_1_ to *b*_2_ if *a*_1_ is a subclass of *b*_1_ but *a*_2_ is not a subclass of *b*_2_:

 
*ω*_1_
  (5) sub1a1,b1∧!sub2a2,b2=>mapa1,a2∧mapb1,b2.
 
*ω*_2_
  (6) !sub1a1,b1∧sub2a2,b2=>mapa1,a2∧mapb1,b2.

Here, *ω*_1_ and *ω*_2_ are negative real-valued weights, rendering alignments that satisfy the formulas possibly but less likely.

*Match Propagation*. Generally speaking, if two concepts *a*_1_ and *a*_2_ match, and there is a relationship *r* between *a*_1_ and *b*_1_ in *O*_1_ and a matching relationship *r*′ between *a*_2_ and *b*_2_ in *O*_2_, then we can increase the probability of match between *a*_2_ and *b*_2_. This is accomplished by adding the following formulas to the model. Formula (7) states that if two classes match, it is more likely that their parent classes match too. Formula (8) describes that if parts of two classes match, it is more likely that the classes match too:

 
*ω*_3_
  (7) sub1a1,b1∧sub2a2,b2∧mapa1,a2=>mapb1,b2.
 
*ω*_4_
  (8) part1a1,b1∧part2a2,b2∧mapa1,a2=>mapb1,b2.

Here, *ω*_3_ and *ω*_4_ are positive real-valued weights, propagating alignment across the structure of ontologies. These formulas capture the influence of the ontology structure and the semantics of ontology relations and increase the probability of matches between entities that are neighbors of already matched entities in the two ontologies. These formulas help to identify correct correspondences and enable deriving missed correspondences based on the hypothesis.

### 3.4. MAP Inference as Alignment Process

After we generate all ground atoms of observed predicates introduced in previous section, we can select an optimal alignment from the incoming hypotheses using MAP inference in Markov logic networks generated by matching formulas. Give two ontologies, we compute the set of ground atoms of the hidden predicates that maximizes the probability given both the ground atoms of observed predicates and the ground formulas. Let *x* be the set of ground atoms of observed predicates and let *y* be the set of ground atoms of hidden predicates *map* with respect to the given ontologies, we compute

(9) $$\begin{array}{c} \underset{y}{\max}\;P\left(\;y\;|\;x\;\right)=\underset{y}{\max}\underset{i}{\sum }\omega_{i}n_{i}\left(\;x,y\;\right), \end{array}$$

where *ω*_*i*_ is the weight of formula *F*_*i*_ and *n*_*i*_(*x*, *y*) is the number of possible worlds where formula *F*_*i*_ holds.

## 4. Results and Discussion

### 4.1. Experimental Setup

We conducted experiments that were implemented in java using the Jena API (jena.apache.org) and SecondString library [14] to create ground atoms and compute the similarity between labels based on Levenshtein distance. Then we applied theBeast [15] for MAP inference in Markov logic networks, using integer linear program (ILP) as base solver. theBeast is a software tool that provides means of inference and learning for Markov logic networks. Experiments were conducted on Fedora 7 with an Intel i5 CPU@3.10 Ghz and 4 GB memory.

We evaluated our model for anatomy ontology matching with thresholds on the similarity *σ* ranging from 0.65 to 0.95. The weights of soft formulas are determined manually. Although the weights for formulas can be learned with an online learner, being able to set qualitative weights manually is crucial as training data is often unavailable. Further, learning weights from reference alignment as training data would lead to results overfitting the data. We set the weights for stability constraints dealing with class hierarchy to −0.01 and set the weight for match propagation to 0.05 based on the consideration that they are reciprocal ideas with stability constraints, hence with roughly equivalent importance.

We evaluated five different settings:

 
*prior*: the formulation includes only a priori confidence.
 
*ca*: the formulation includes a priori confidence and cardinality constraints.
 
*ca + co*: the formulation includes a priori confidence, cardinality, and coherence constraints.
 
*ca + co + st*: the formulation includes a priori confidence, cardinality constraints, coherence constraints, and stability constraints.
 
*ca + co + st + mp*: the formulation includes a priori confidence, cardinality constraints, coherence constraints, stability constraints, and matching propagation.

### 4.2. Experimental Results

We use* precision*,* recall*, and* F-measure* to measure the performance of the matching results. Given the reference alignment, we compute the* precision* as the number of correct correspondences over the total number of correspondences in the computed alignment. We compute the* recall* as the number of correct correspondences over the number of correspondences in the reference alignment. Then, we compute the* F-measure* as

(10) $$\begin{array}{c} F\text{-}measure=2*precision*\frac{recall}{\left(precision+recall\right)}. \end{array}$$

**Figure 3:**
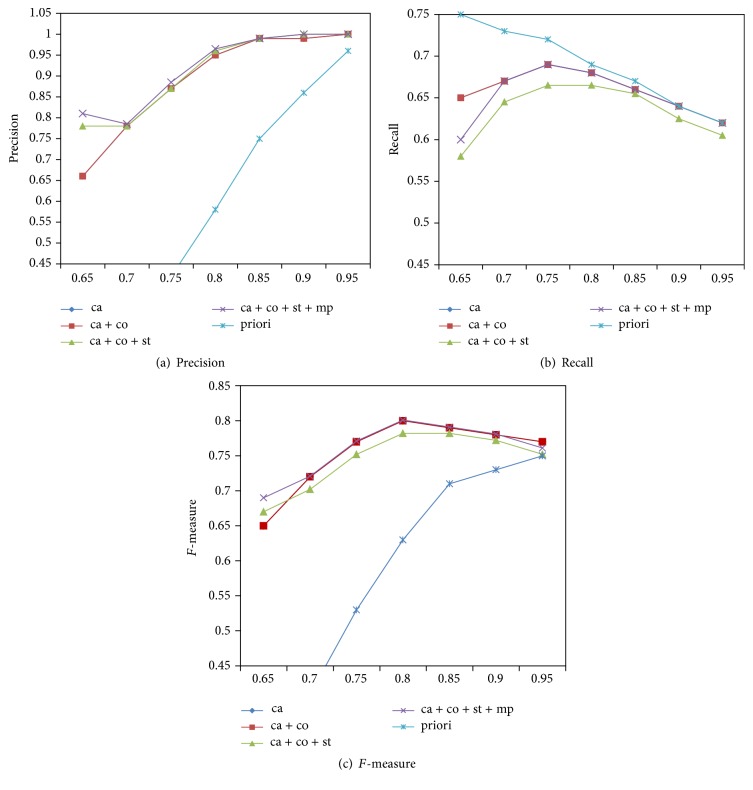
Results for thresholds ranging from 0.65 to 0.95.

Figure 3 compares* precision*,* recall*, and* F-measure* scores of generated alignments over the reference alignment for thresholds ranging from 0.65 to 0.95 under different settings. From Figure 3, we can see that our method achieves the highest precision in the setting of* ca + co + st + sp*, while achieving the highest recall in the setting of* priori*. We obtain significant improvement on *F*-measure when adding more matching formulas into the model. We also note that there is no obvious difference between* ca* and* ca + co*. It is because only the human anatomy ontology defines the relationships of disjointWith. However, we keep coherence constraints in our model since it can further improve the quality of results if the relationships of disjointWith were added into the mouse anatomy ontology in the future. Overall, the precision increases with the growth of the threshold, while the recall slightly decreases for higher thresholds in various settings. The margins between different settings become smaller for higher thresholds than for lower thresholds. It is because there is only a small number of incorrect correspondences in candidates when we apply a threshold greater than 0.8. We achieve the maximum *F*-measure score at threshold 0.8.

We manually sample several false positive correspondences and false negative correspondences to analysis. We found that false positive correspondences were mainly caused by similar labels in spelling. For example, false correspondence (“NCI_C33592”, “MA_0002058”) has similar labels of “Spiral_Artery” and “sural artery.” Furthermore, the superclass of “NCI_C33592” (“NCI_C12372”) and the superclass of “MA_0002058” (“MA_0002058”) happen to be matched, while false positive correspondences were mainly caused by the dissimilarity of labels, such as “Tarsal_Plate” for “NCI_C33736” and “eyelid tarsus” for “MA_0000270.” And “NCI_C33736” has no subclass and subpart; hence we cannot find the correspondence through formula (7) or (8).

**Figure 4:**
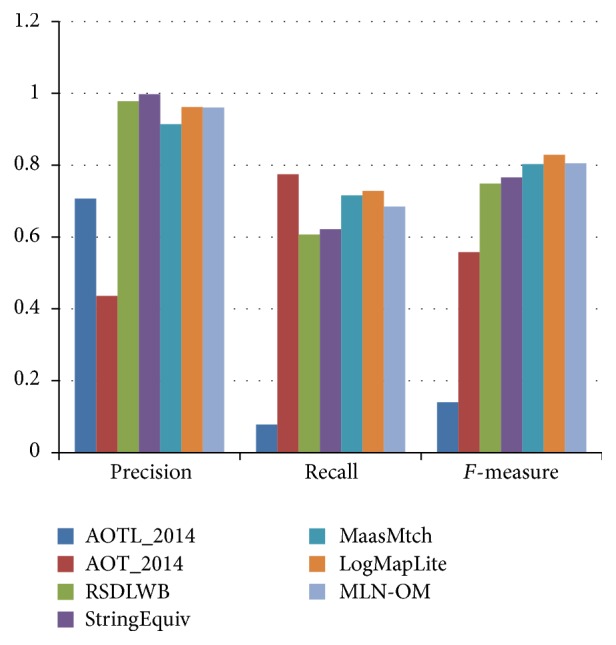
Comparing with results of OAEI 2014.

Figure 4 is a comparison of the performance of our method and participating systems of OAEI 2014 which also produce coherent alignment in anatomy track. From Figure 4, we can see that our method (MLN-OM) outperforms most of systems and is comparable with the best system (LogMapLite). Notice that we use a simple similarity measure based on Levenshtein distance in pruning phase and focus on the Markov logic model for ontology matching, while LogMapLite uses an external lexicon (e.g., WordNet or UMLS-lexicon) in the phase of computing an initial set of equivalence anchor mappings, which can be easily adopted by our method in the pruning phase to further improve the quality of matching results.

## 5. Conclusions

In this paper, we propose a Markov logic model for anatomy ontology matching. The model combines five types of matching strategies, namely, a priori confidences, cardinality constraints, coherence constraints, stability constraints, and match propagation. Experimental results demonstrate the effectiveness of the proposed approach.

## Figures and Tables

**Table 1:** Core predicates for anatomical ontology matching.

	Predicate	Description
Observed	*class*_*i*_(*c*)	*c* is a class from ontology *O*_*i*_, *i* ∈ {1,2}
	*label*_*i*_(*c*, *l*)	Class *c* has a label *l*
	*sub*_*i*_(*a*, *b*)	*a* is a subclass of *b*
	*part*_*i*_(*a*, *b*)	*a* is a part of *b*
	*dis*_*i*_(*a*, *b*)	*a* is disjoint with *b*
	*sim*(*l*_1_, *l*_2_, *σ*)	Labels *l*_1_ and *l*_2_ are similar to a similarity of *σ*

Hidden	*map*(*c*_1_, *c*_2_)	Class *c*_1_ from *O*_1_ corresponds to class *c*_2_ from *O*_2_
